# Genome-Wide Characterization of the *sHsp* Gene Family in *Salix suchowensis* Reveals Its Functions under Different Abiotic Stresses

**DOI:** 10.3390/ijms19103246

**Published:** 2018-10-19

**Authors:** Jianbo Li, Jin Zhang, Huixia Jia, Zhiqiang Yue, Mengzhu Lu, Xuebing Xin, Jianjun Hu

**Affiliations:** 1 State Key Laboratory of Tree Genetics and Breeding, Chinese Academy of Forestry, Beijing 100091, China; Lijb2017@caf.ac.cn (J.L.); zhang007jin@163.com (J.Z.); huixia__jia@126.com (H.J.); yue7210@sina.com (Z.Y.); lumz@caf.ac.cn (M.L.); 2 Experimental Center of Forestry in North China, Chinese Academy of Forestry, Beijing 102300, China; 3 Key Laboratory of Tree Breeding and Cultivation of National Forestry and Grassland Administration, Research Institute of Forestry, Chinese Academy of Forestry, Beijing 100091, China; 4 Collaborative Innovation Center of Sustainable Forestry in Southern China, Nanjing Forestry University, Nanjing 210037, China

**Keywords:** small heat shock proteins (sHsp), phylogenetic analysis, gene structure, expression analysis, *Salix suchowensis*

## Abstract

Small heat shock proteins (sHsps) function mainly as molecular chaperones that play vital roles in response to diverse stresses, especially high temperature. However, little is known about the molecular characteristics and evolutionary history of the *sHsp* family in *Salix suchowensis,* an important bioenergy woody plant. In this study, 35 non-redundant *sHsp* genes were identified in *S. suchowensis*, and they were divided into four subfamilies (C, CP, PX, and MT) based on their phylogenetic relationships and predicted subcellular localization. Though the gene structure and conserved motif were relatively conserved, the sequences of the Hsp20 domain were diversified. Eight paralogous pairs were identified in the *Ssu*-*sHsp* family, in which five pairs were generated by tandem duplication events. *K*a/*K*s analysis indicated that *Ssu*-*sHsps* had undergone purifying selection. The expression profiles analysis showed *Ssu-Hsps* tissue-specific expression patterns, and they were induced by at least one abiotic stress. The expression correlation between two paralogous pairs (*Ssu*-*sHsp22*.*2*-*CV*/*23*.*0*-*CV* and *23*.*8*-*MT*/*25.6*-*MT*) were less than 0.6, indicating that they were divergent during the evolution. Various *cis*-acting elements related to stress responses, hormone or development, were detected in the promoter of *Ssu*-*sHsps*. Furthermore, the co-expression network revealed the potential mechanism of *Ssu*-s*Hsps* under stress tolerance and development. These results provide a foundation for further functional research on the *Ssu*-*sHsp* gene family in *S. suchowensis*.

## 1. Introduction

Plants are often exposed to complex environmental stimuli, so they inevitably suffer various stresses including extreme temperature, flooding, drought, and salinity, leading to restriction of their growth or even cause death. As a sessile organism, plants have evolved sophisticated mechanisms to accommodate these stresses over long lifespans [1]. Heat shock proteins (Hsps) function as molecular chaperones that are involved in the folding, localization, repairing, and degradation of protein molecules in all living organisms and play vital roles in response to diverse stresses [2].

Hsps can be divided into five major groups, including Hsp100, Hsp90, Hsp70, Hsp60, and small Hsp (sHsp)/Hsp20 according to their protein molecular weight [3]. The monomer molecular weight of sHsp ranges from 15 to 42 kDa. As molecular chaperones, sHsp perform functions in an ATP-independent manner through forming a complex with the denatured proteins to avoid protein degradation [4]. On the basis of the subcellular localization, sHsps are divided into five subfamilies: cytosol (C) subfamilies (CI-CVI) are located in the cytoplasm or nucleus; mitochondria (M) subfamilies (MTI and MTII) are located in the mitochondria; the endoplasmic reticulum (ER), chloroplast (CP), and peroxisome (PX) subfamilies are located in the endoplasmic reticulum, chloroplast, and peroxisome, respectively [5]. The essential characteristic of sHsp is the presence of a highly conserved 80–100 amino acid α-crystallin domain (ACD)/Hsp20 domain which are located in the C-terminal region. This domain is composed of a series of β-strand structures and can be divided into three parts, N-terminal consensus region I (CRI) with β2-β3-β4-β5, C-terminal consensus region II (CR II) with β7-β8-β9, and a hydrophobic region loop (β6-loop) that connects CRI and CRII [5].

To date, *sHsps* have been identified in various organisms from lower eukaryotes to higher plants and animals, two *sHsps* were identified in *Saccharomyces cerevisiae* [6], 10 *sHsps* in human [7], and 13 *sHsps* in zebrafish [8], while 19 *sHsps* in *Arabidopsis thaliana* [9], 23 in rice (*Oryza sativa*) [10], 27 in wheat (*Triticum aestivum*) [11], 35 in pepper (*Capsicum annuum*) [12], and 37 in *Populus trichocarpa* [13]. This indicates that the number of *sHsps* in the sessile plant species are higher than that in bacteria and animals. The expansion levels of the *sHsp* family is positively correlated with their diversification and adaptation to complicated stress conditions in high plants. In addition, the functions of several *sHsps* have been investigated based on genetic evidence. The expression of *sHsp24*.*4* from *Musa accuminata* is induced under high temperature, and its overexpression can increase tolerance against heat stress in the transgenic tomato [14]. Heterologous expression of rice *OsHsp26* enhances tolerance against oxidative and heat stresses possibly through protecting photosystem II in transgenic tall fescue [15]. Transgenic *Arabidopsis* plants with overexpression of poplar *PtsHsp17.8* show higher tolerance to heat and salt stresses than control plants through increasing antioxidative enzyme activities [16]. After ectopically expressing *NnHsp17*.*5* from *Nelumbo nucifera* in *Arabidopsis*, transgenic seeds exhibit increased superoxide dismutase activity after accelerated aging treatment [17]. In addition, the function of *sHsp17*.*3* from *Juglans regia* [18], *sHsp17.7* from *Vaccinium corymbosum* [19], and *sHsp22*.*5* from *Capsicum annuum* [20] were proved to be involved in extreme temperature and salt stresses. All these studies have indicated that *sHsps* are involved in stress responses and play important roles in plant adaptation to various environments.

Willow (*Salix*) is a member of *Salicaceae* family and it is regarded as a promising economic plant that can be used as renewable biomass for bioenergy, because of its easily vegetative propagation, rapid growth, short-rotation, and substantial biomass yields [21]. As protective proteins, *sHsps* in willow have attracted attention for their potential roles against stresses. However, systematic research on the *sHsp* family in willow is still lacking. *Salix suchowensis* is a special shrub willow species that is found in the north of China [22]. The completion of the genome sequence of *S*. *suchowensis* make it possible to investigate the *Ssu*-*sHsp* gene family [23]. Nowadays, increasing public data (genomic, transcriptomic, or proteomic data) provide abundant information to help understand gene function, like sHsp in poplar [13], *Arabidopsis thaliana* [9], rice (*Oryza sativa*) [10], etc. In the present study, 35 genes encoding Ssu-sHsp proteins were identified in the *S*. *suchowensis* genome using bioinformatics methods. We analyzed their chromosomal localization, genes and protein structure, and duplication events. Subsequently, the expression profiles of all *Ssu-sHsp* genes were detected in various organs/tissues and under different abiotic stresses (heat, drought, cold, salt, and abscisic acid (ABA) treatment) to explore their potential functions in stress responses. The *cis*-acting elements and co-expression network were also analyzed to investigate the possible regulation mechanisms of *Ssu*-*sHsps*. Our analysis lays the foundation for the further characterization of *Ssu*-*sHsp* genes and genetic improvement of *S*. *suchowensis* resistance to stress conditions.

## 2. Results

### 2.1. Genome-Wide Identification and Phylogenetic Analysis of Ssu-sHsp in S. suchowensis

To identify *sHsp* genes in *S. suchowensis*, the protein sequences of 19 *A. thaliana* sHsp (AtsHsp) [9] and 37 *P*. *trichocarpa* sHsp (PtsHsp) [13] were used as a query to perform basic local alignment search tool algorithms (BLASTP) and Hidden Markov models (HMMs) search against the *S. suchowensis* genome database [23]. A total of 35 non-redundant Ssu-sHsp proteins were identified, and the information of open reading frame length, protein length, molecular weight (Mw), and isoelectric point (pI) are shown in Table 1. The length of proteins varied from 116 to 350 amino acids, the predicted pI ranged from 4.60 to 9.65, and the Mw from 13.3 to 38.7 kDa (Table 1 and Table S1).

**Figure 1:**
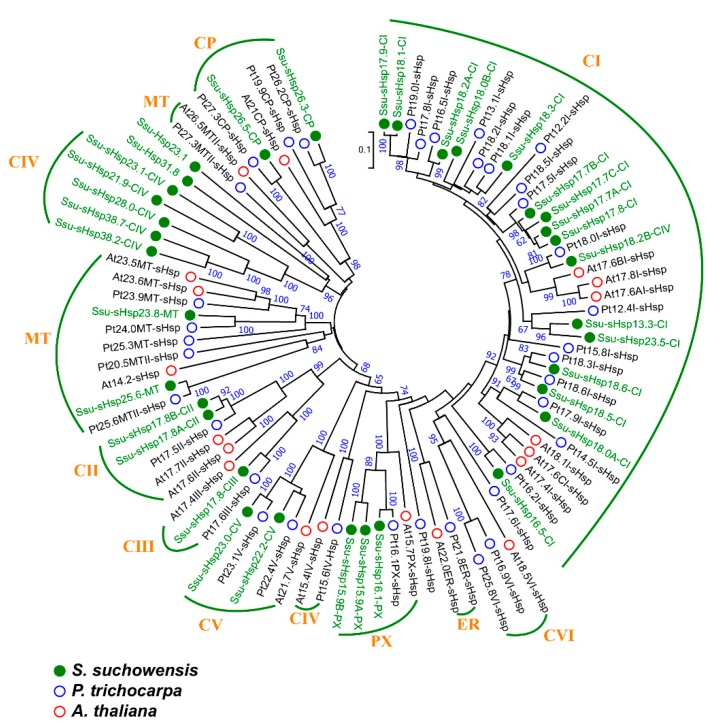
Phylogenetic relationships of sHsp family members from *Salix suchowensis*, *Populus trichocarpa,* and *Arabidopsis thaliana*. Multiple alignment was performed using Clustal X2.1, and the phylogenetic tree was constructed using MEGA 6.06 by the neighbor-joining (NJ) method with 1000 bootstrap replicates. s*Hsp* from *S. suchowensis*, *P*. *trichocarpa,* and *A. thaliana* were marked with green, blue, and red circles, respectively.

A phylogenetic tree was constructed using the full-length protein sequences of sHsp from *S*. *suchowensis*, *A*. *thaliana,* and *P*. *trichocarpa* to investigate their evolutionary relationship. In *S. suchowensis*, the sHsp family was mainly classified into four subfamilies, including C (cytosol), CP (chloroplast), PX (peroxisome), and MT (mitochondria), but lacked the endoplasmic reticulum (ER) subfamily in comparison with AtsHsps and PtsHsps [9,13]. The C subfamily of Ssu-sHsp, which had 26 members, was subdivided into five groups (CI-CV): 16 members in CI, two members in CII and CV, one member in CIII, and five members in CIV, respectively; the CP and MT subfamilies individually contained two members; three members were existed in PX subfamily (Table 1 and Figure 1). On the basis of their phylogenetic relationships, potential subcellular localization/group, and their Mw, we named the Ssu-sHsps the “Ssu-sHsp (Mw)-(group)”. For example, the Ssu-sHsp member willow_GLEAN_10019104 with Mw 16.5 kD belonging to the CI group was named Ssu-sHsp16.5-CI (Table 1 and Figure 1).

### 2.2. Gene Structure and Protein Domain Analysis of Ssu-sHsps

**Figure 2:**
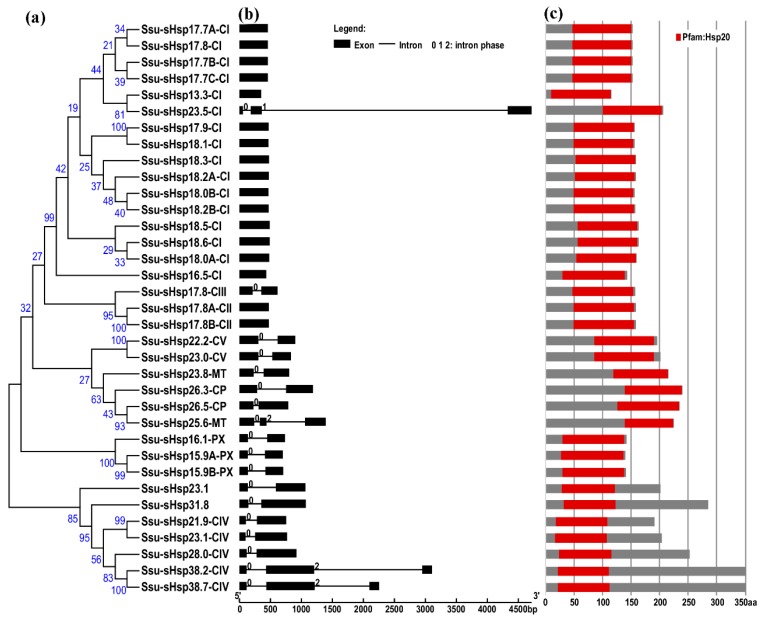
Phylogenetic relationships, gene structure, and protein structures of the *sHsp* genes in *Salix suchowensis*. (**a**) Multiple alignment of Hsp20 domain was executed using Clustal X2.1 and a phylogenetic tree was constructed using MEGA 6.06 by the neighbor-joining (NJ) method with 1000 bootstrap replicates. (**b**) The exon/intron structures of the *Ssu*-*sHsps* genes. Boxes represent exons and lines represent introns. The numbers indicate the splicing phases of the *Ssu-sHsp*s: 0, phase 0; 1, phase 1; and 2, phase 2. (**c**) The protein structures of the Ssu-sHsp proteins. Red boxes represent the Hsp20 domain.

To gain further insight into the structural diversity of different groups of *Ssu*-*sHsp* genes, the simplified neighbor-joining (NJ) phylogenetic tree of their Hsp20 domains was constructed, as well as the analysis of gene structures and functional domain (Figure 2). As shown in Figure 2a, all of the Ssu-sHsp members still remained in the same subfamily as that used by the full-length protein sequences. All the members of CI (except *Ssu*-*sHsp23*.*5*-*CI*) and CII groups were intronless, four *Ssu*-*sHsps* (*23.5*-*CI*, *25*.*6*-*MT*, *38*.*2*-*CIV* and *38*.*7*-*CIV*) were comprised of two introns, and the other 14 *Ssu*-*sHsps* contained one intron. While the *Ssu*-*sHsps* with two-introns showed phase 0 in the first intron, but the second intron of *Ssu*-*sHsp23*.*5*-*CI* showed phase 1 and others *Ssu*-*sHsp* (*25*.*6*-*CP*, *38*.*2*-*CIV* and *38*.*7*-*CIV*) showed phase 2 in the second intron. These results indicated that the members of the same subfamily shared similar gene structures and intron phases (Figure 1 and Figure 2).

**Figure 3:**
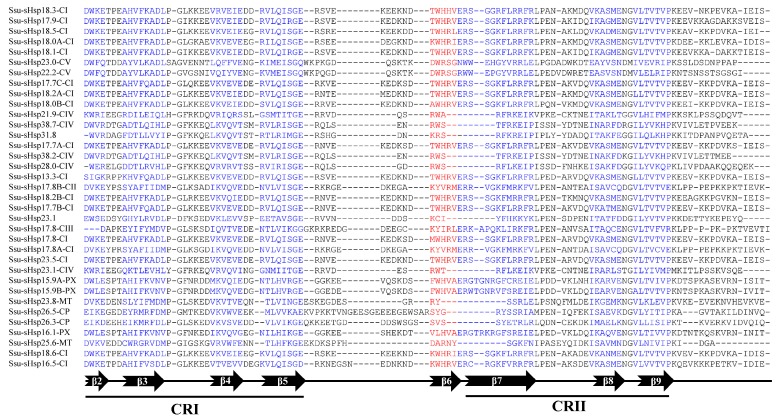
Multiple sequence alignment of the Hsp20 domains of the Ssu-sHsp proteins.

**Figure 4:**
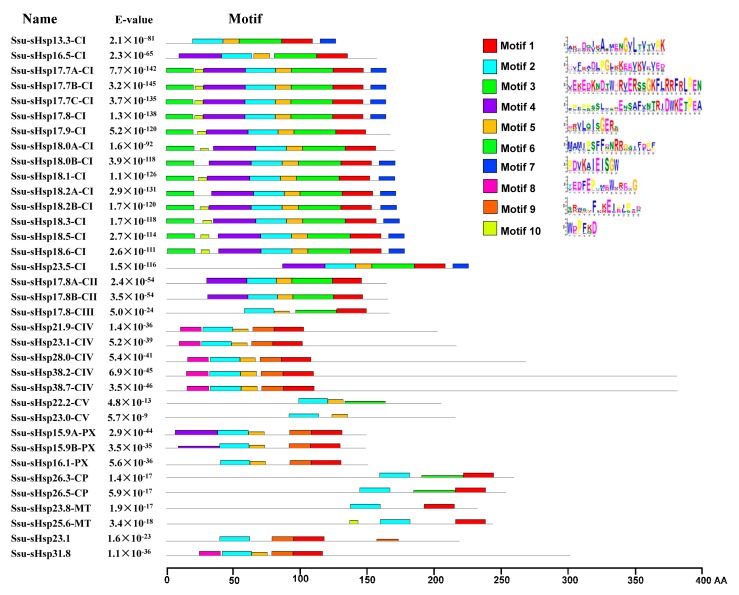
Distribution of conserved motifs in the Ssu-sHsp proteins. The motifs were identified by MEME. Different motifs are indicated by different colored number 1–10.

The analysis of the functional domain showed that all the Ssu-sHsp proteins have the Hsp20 domain, but their location was different and there existed two types: the Hsp20 domain of Ssu-sHsp23.1, Ssu-sHsp31.8, and the members in CIV subgroup were located near the N-terminal, while the Hsp20 domain of the others near the C-terminal (Figure 2c). Multiple sequence alignment was conducted to identify the variations in Hsp20 domains. As shown in Figure 3, the Hsp20 domain in most of Ssu-sHsps was composed by CRI, CRII, and β6-loop, which was consistent with the report in switchgrass [24] and pepper [12]. However, Ssu-sHsp17.8-CIII lacked the β2, and ten *Ssu-sHsps* (*21.9-CIV, 23.1*, *23.1-CIV*, *26.3-CP*, *26.5-CP*, *23.8-MT*, *28.0-CIV*, *31.8*, *38.2-CIV*, and *38.7-CIV*) lacked the β6-loop and contained incomplete β7 (Figure 3). After searching with the MEME program, ten conserved motifs were identified in Ssu-sHsps (Figure 4). Thereinto, the CRI contained the motifs 2, 4, and 5, while CRII contained motifs 1 and 3. However, only 19 of the 35 Ssu-sHsp proteins contained these five motifs, revealing the diversification of Hsp20 domain in *S. suchowensis* sHsps (Figure 4).

### 2.3. Expansion of the sHsp Family in S. suchowensis

**Figure 5:**
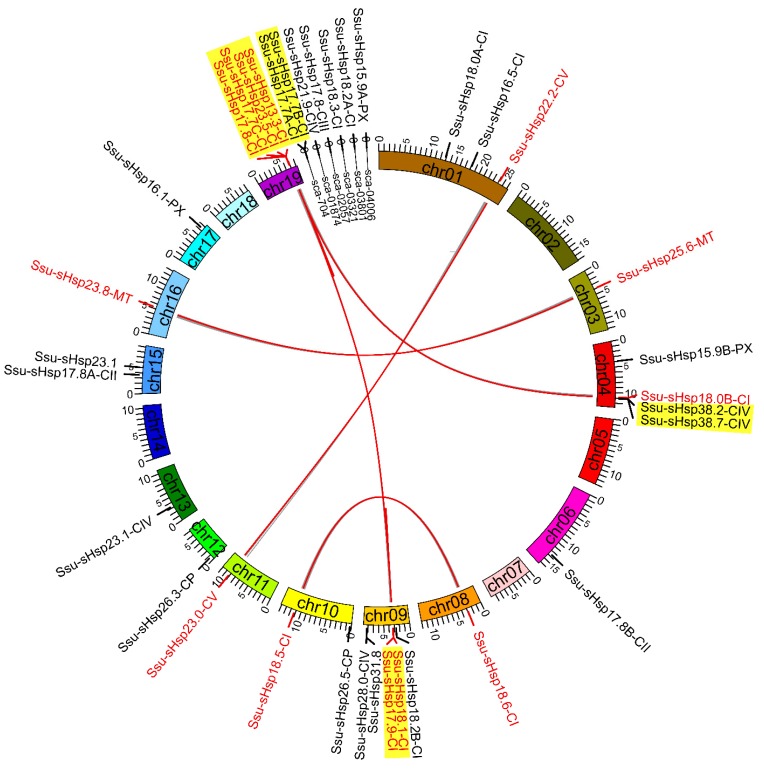
Chromosomal location and duplication event analyses of *Ssu*-*sHsps* in the *S. suchowensis* genome*.*

To analyze the expansion and genomic distribution of *Ssu*-*sHsp* genes, the chromosomal location and duplication events of the *Ssu*-*sHsp* family were analyzed based on the current genomic information from the *S. suchowensis* genome database. The 35 *Ssu*-s*Hsp* genes were unevenly mapped onto 14 chromosomes and six scaffolds in the *S. suchowensis* genome (Figure 5). Chr09 contained the largest number of five *Ssu*-*sHsps*, followed by Chr04 and Chr19 that each contained four *Ssu*-*sHsps* (Figure 5 and Table 1). On the basis of the chromosomal location, phylogenetic relationships, and gene structures, eight paralogous pairs were identified in the *Ssu*-*sHsp* family (Table 2). Among of them, three paralogous pairs (*Ssu*-*sHsp18*.*5*-*CI*/*18*.*6*-*CI*, *22*.*2*-*CV*/*23*.*0*-*CV*, *25*.*6*-*MT*/*23*.*8*-*MT*) were generated by whole genome duplication (WGD) events and another five pairs (*Ssu*-*sHsp17*.*7A*-*CI*/*17*.*7B*-*CI*, *17*.*8*-*CI*/*17*.*7C*-*CI*, *18*.*1*-*CI*/*17.9*-*CI*, *23*.*5*-*CI*/*13*.*3*-*CI*, *38*.*2*-*CIV*/*38*.*7*-*CIV*) were generated by tandem duplication events (Figure 3 and Table 2). The substitution ratio of non-synonymous (*K*a)/synonymous (*K*s) was used to measure the selection pressures for duplicated gene pairs [25]. In our study, the *K*a/*K*s ratios of all the eight *Ssu*-*sHsp* paralogous pairs were less than 1, suggesting that these *Ssu*-*sHsp* paralogous pairs have evolved from purifying selection (Table 2).

### 2.4. Expression Patterns of Ssu-sHsps across Tissues and under Various Stresses

**Figure 6:**
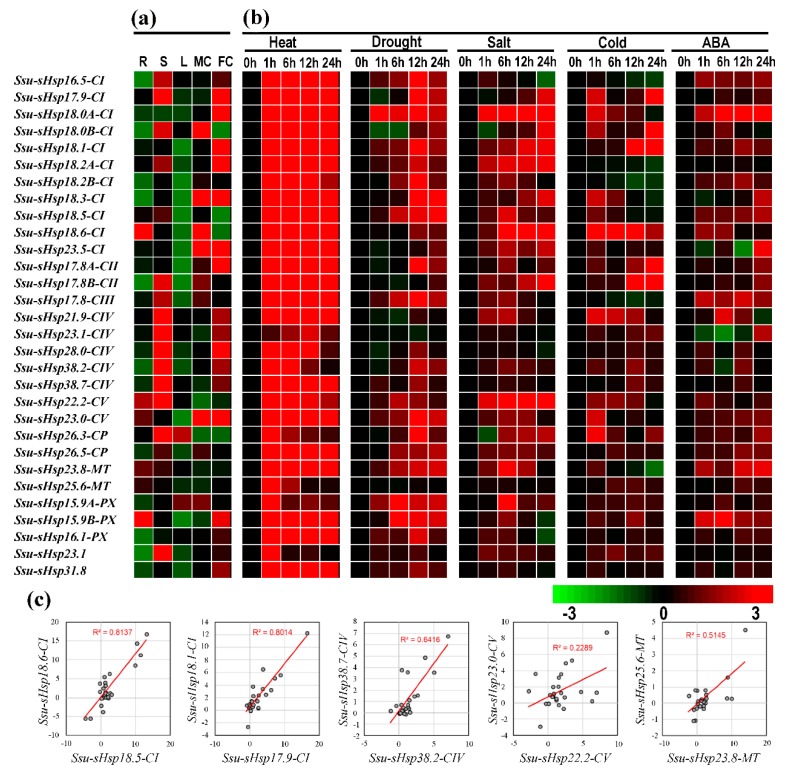
Expression profiles of *Ssu-sHsps* by qRT-PCR. (**a**) Expression analysis of *Ssu*-*sHsps* in five tissues: root (R), stem (S), leaf (L), female catkin (FC), and male catkin (MC). The expression of *Ssu*-*sHsp* in different tissues were compared with the median value among these tissues. (**b**) Expression analysis of *Ssu*-*sHsp* genes under various abiotic stresses after treatments at 1, 6, 12, or 24 h under heat (37 °C), drought (20% *w*/*v* PEG 6000), salt (150 mM NaCl), cold (4 °C), and 100 µM abscisic acid (ABA) treatment. The different colors represent log2 transformed values compared with control (0 h). (**c**) Correlation of expression of five *Ssu*-*sHsp* paralogous pairs across various tissues and under stresses. The *x*- and *y*-axis indicate expression levels across various tissues and under stresses in each paralogous pair. For example, the *x*-axis represents *Ssu-sHsp18.5-CI* and *y*-axis represents *Ssu-sHsp18.6-CI* in pair *Ssu*-*sHsp18.5-C*I/*Ssu-sHsp18.6*-*CI*.

In order to gain the information about the spatial and temporal expression pattern of *Ssu*-*sHsp*s, qRT-PCR was performed to explore the expression levels of *Ssu*-*sHsps* in five different tissues including root (R), stem (S), leaf (L), female catkin (FC), and male catkin (MC). As illustrated in Figure 6a, the majority of *Ssu*-*sHsps* have special tissue expression patterns. Four *Ssu*-*sHsps* (*18*.*6*-*CI*, *15*.*9B*-*PX*, *23*.*8*-*MT,* and *25*.*6*-*MT*) were predominantly expressed in the root. Thirteen *Ssu*-*sHsps* (*16*.*5*-*CI*, *18*.*5*-*CI*, *17*.*8B*-*CIII*, *17*.*8*-*CIII*, *21*.*9*-*CIV*, *23*.*1*-*CIV*, *28*.*0*-*CIV*, *38*.*2*-*CIV*, *38*.*7*-*CIV*, *22*.*2*-*CV*, *26*.*3*-*CP*, *26*.*5*-*CP,* and *23*.*1*) were highly expressed in the stem. Thirteen *Ssu*-*sHsps* (*18*.*0B*-*CI*, *18*.*3*-*CI, 17*.*8B*-*CII*, *15*.*9A*-*PX, 17*.*9*-*CI*, *18*.*0A*-*CI*, *18*.*1*-*CI*, *18*.*2A*-*CI*, *23*.*5*-*CI*, *17*.*8A*-*CII*, *23*.*0*-*CV, 16*.*1*-*PX,* and *31*.*8*) displayed relatively high expression levels in the reproductive organs (female catkin or male catkin). Notably, the expression levels of all the *Ssu*-*sHsp* genes were relatively low in the leaf across the five analyzed tissues (Figure 6a).

**Figure 7:**
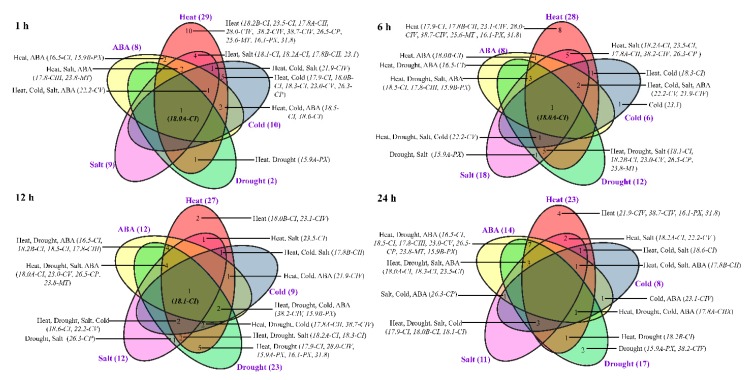
Different stress response model of *Ssu-sHsp* genes at different times after treatments.

To investigate the responses of *Ssu*-*sHsps* to various environmental stresses, the expression profiles of *Ssu*-*sHsps* were detected using qRT-PCR under heat, drought, salt, cold, and ABA treatments. Among the detected 30 *Ssu*-*sHsps*, 29 *Ssu*-*sHsps* were promptly and dramatically up-regulated after heat treatments for 1 h (Figure 6b and Figure 7), and most of them maintained high expression levels until the end (24 h) of the heat stress (Figure 6b and Figure 7). Aside from heat stress, several *Ssu*-*sHsps* (e.g., *Ssu*-*sHsp18*.*0A*-*CI*, *18*.*1*-*CI*, *18*.*2A*-*CI*, *22*.*2*-*CV,* and *15*.*9A*-*PX*) were responsive to both drought and salt stresses. In contrast to the large number of *Ssu*-*sHsps* strongly induced by heat, drought, salt, and ABA stresses, only six, nine, and eight *Ssu*-*sHsps* were responsive to cold treatment at 6, 12 and 24 h, respectively. It was noted that *Ssu*-*sHsp18.0A*-*CI* were significantly responsive to all the tested treatments at 1 h and 6 h of treatment (Figure 6 and Figure 7).

Among the eight pairs, the expression of five *Ssu*-*sHsp* paralogous pairs (*18*.*5*-*CI*/*18*.*6*-*CI*, *17*.*9*-*CI*/*18.1*-*CI*, *38*.*2*-*CIV*/*38*.*7*-*CIV*, *22*.*2*-*CV*/*23*.*0*-*CV,* and *23*.*8*-*MT*/*25.6*-*MT*) were detected successfully. Only three *Ssu-sHsp* pairs (*Ssu*-*sHsp18*.*5*-*CI*/*18*.*6*-*CI*, *17.9-CI*/*18.1-CI*, and *38.2-CIV*/*38.7-CIV*) showed highly similar patterns with *R*^2^  >  0.6 across various tissues and stresses, while the other two detected paralogous pairs showed divergent expression (Figure 6c).

### 2.5. Characterization of Cis-acting Elements in the Promoter Regions of Ssu-sHsps

**Figure 8:**
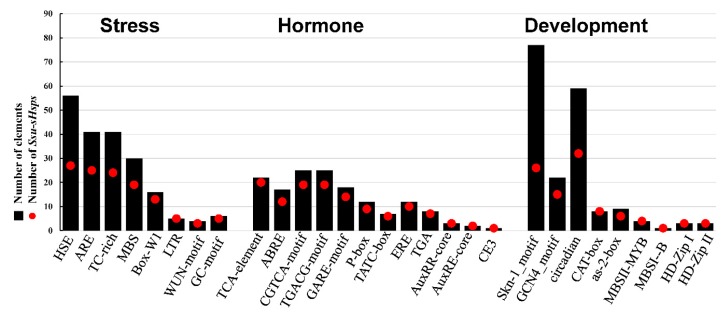
Various *cis*-acting elements in the promoter of *Ssu-sHsp* genes.

To further investigate the possible regulation mechanism of *Ssu*-*sHsps* in *S. suchowensis*, the *cis*-elements in the promoter regions of *Ssu*-*sHsps* were analyzed using the PlantCARE database. A series of stress-related (e.g., heat, drought, low temperature, and anaerobic induction), hormone-related (e.g., ABA, SA, GA, and MeJA), and development-related (e.g., endosperm expression, circadian control, meristem expression, and light response) *cis*-acting elements were identified (Figure 8 and Figure S1). In stress-related *cis*-acting elements, 77.1% of *Ssu*-*sHsps* (27 genes) had the HSE (heat responsive elements) which was the most abundant *cis*-acting element with its number reaching 56. There were 41 ARE (essential for the anaerobic induction) and 41 TC-rich repeats (stress responsiveness) that were located in 12 and 10 *Ssu*-*sHsp* promoters, respectively. In hormone-related *cis*-acting elements, TCA-element (salicylic acid responsiveness), ABRE (abscisic acid responsiveness), CGTCA-motif (MeJA-responsiveness), and GARE-motif (MeJA-responsiveness) were identified in the promoters of 20, 11, 19, and 19 *Ssu*-*sHsps*, respectively. Furthermore, large numbers of *cis*-acting elements related to endosperm expression (Skn-1) and circadian (GCN4_motif and circadian *cis*-acting element) were detected, implying that *sHsp* might be also involved in developmental processes in *S. suchowensis*.

### 2.6. The Co-expression Network of Ssu-sHsps

**Figure 9:**
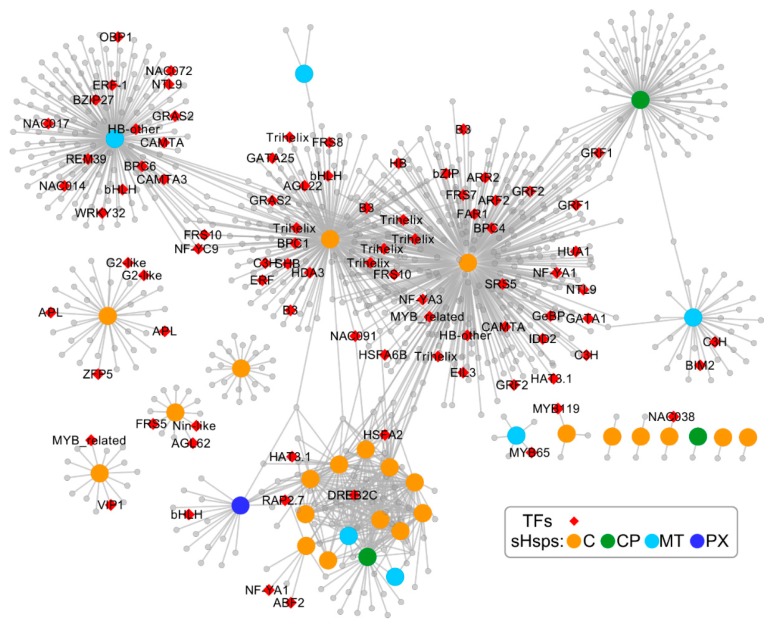
Co-expression network of *sHsp* gene family. Orange, green, cyan, and blue nodes represent C, CP, MT, and PX subfamily members, respectively; red diamond nodes represent transcription factors; gray nodes are other co-expressed genes. The functional descriptions of these transcription factors are listed in Table S2.

To further predict the relationship between *Ssu*-s*Hsp* genes and other genes, a co-expression network of *sHsp* genes was constructed according to their orthologous in *P. trichocarpa* (Figure 9). The *Ssu*-*sHsps* lacked an ER subfamily compared with the *PtsHsp* family, and *Ssu*-*sHsp* did not correspond one-to-one with *P*. *trichocarpa* members (Figure 1). Thus, the ER members of *PtsHsps* were removed in the co-expression network, and the subfamily was used as a unit to study the co-expression relationship. In this network, 970, 235, 116, and 20 genes were co-expressed with *sHsp* in the C, CP, MT, and PX subfamily, respectively. Moreover, some of them shared the same co-expressed genes. It was noted that the network contained a transcription factor which related to stress response (e.g., *HSFA6B*, *HSFA2*, *DREB2C*), development (e.g., *GRF*, *Floral homeotic protein HUA1*, *AGAMOUS*-*like 62*), and hormone (e.g., *ARF2*, *ARR2* and *AREB3*), and several of them were hub genes (Figure 9 and Table S2). This indicated that the *sHsps* in *S*. *suchowensis* might be function in stress tolerance or developmental processes through cooperation with specific transcription factors or other functional genes.

## 3. Discussion

As ubiquitous molecular chaperones, sHsps are present in various organisms and play prominent roles in alleviating injuries from diverse abiotic and biotic stresses, especially in heat stress [26]. The features and functions of the sHsp family have been studied in many model plants (e.g., *Arabidopsis*, rice, and poplar) [9,10,13,27], however, very little is known regarding *sHsp* genes in *S. suchowensis*, which is regarded as a short-rotation woody biomass for bioenergy sources and biofuel.

In this study, 35 *Ssu-sHsp* genes were identified in the *S. suchowensis* genome, which is less than that in *P*. *trichocarpa* (37 *PtsHsps*), and this number is consistent with the relative genome size of these two specie (~425 Mb of *S. suchowensis* and ~485 Mb of *P*. *trichocarpa*) [13]. However, no ER-family member was identified, possibly because of the incomplete genome assembly of *S. suchowensis*. Gene duplication including WGD and tandem duplication events play a crucial role in expansions of the gene family during species evolution [28]. In the *Ssu*-*sHsp* gene family, eight paralogous pairs were identified, including three and five pairs which were generated by WGD and tandem duplication events, respectively, and contributing to the expansion of the CI (five pairs), CIV (one pair), CV (one pair), and MT (one pair) subgroups. Among of them, CI was the largest subgroup containing most expansion genes, which was consistent with reports in other plants [29]. The duplicated gene pairs shared high amino acid sequence identity and similar gene structures, but the expression patterns of duplicated gene pairs (*Ssu*-*sHsp18*.*5*-*CI*/*18*.*6*-*CI*, *22*.*2*-*CV*/*23*.*0*-*CV*) were different across various tissues and under stresses, and they kept a lower correlation with *R*^2^ < 0.6 (Figure 6) indicating that their function might be divergent during the evolution.

The investigation of gene organization showed that 31 *Ssu*-*sHsp* genes (~88.5%) contained one or no intron and confirmed that *sHsps* have less introns in higher plants. Its reason might be that the length of sHsp itself is too short. It has been reported that the intron number likely affects the efficiency of transcription [30]. When comparing the relationship between the intron numbers and their expression patterns of *Ssu*-*sHsps* under stress conditions, we found less introns is helpful for *Ssu*-*sHsps* to prompt induction under stresses (Figure 6). Therefore, the gene structure might also play roles in controlling its transcriptional level under stress condition [31].

In the native state, sHsps always exist as multimeric complexes with variable numbers of subunits [5]. The Hsp20 domain is crucial for the formation of multimeric complexes that are important for their chaperone activity [4]. In our study, all the Ssu-sHsp proteins contained the Hsp20 domain, but multiple sequence alignments and motifs analysis showed that the sequences of Hsp20 were diverse among the Ssu-sHsp family and even parts of them lacked the β2-strand, β6-loop, or the β7-strand (Figure 3 and Figure 4). It is reported that the β2-strand is associated with structure dimerization [11,26]. Additionally, the β6-loop that links the β5-strand and β7-strand is related to dimer formation [32]. Thus, the Ssu-sHsp proteins lacking β2, β6, or β7 might be loss-of-function, or even leading to functional diversity which will need to be fully elucidated.

In the co-expression network, floral development-related genes (*AGAMOUS*-*like 62*, *MADS*-*box,* and *Floral homeotic protein HUA1*) were co-expressed with C subfamily members (Figure 9 and Table S2). In our study, 14 *Ssu*-*sHsps* had relatively high expression levels in reproductive organs, and ten of them belonged to the C subfamily (Figure 6a), in which most members were induced by at least one or two stresses (Figure 6b and Figure 7). During the reproductive growth, flowers are always sensitive to high temperature and other environmental stresses [33,34]. Therefore, it can be inferred that *Ssu*-*sHsp* with high expression levels in reproductive organs might be cooperating with the flower development-related genes in regulating flower development and maintaining the cellular homeostasis between stress and normal conditions. In addition, 13 *Ssu*-*sHsps* were detected as being highly expressed in the stem, a similar result is also reported in poplar [13]*.* The stem is the product of continuous division and differentiation of the cambium cells in the secondary growth. In addition, the activity of the cambium is susceptible to the external environment [35]. Considering the stem-specific expression patterns of *Ssu*-*sHsps* and their conserved function, they might be involved in adjustment stem development by protecting the protein folding which plays roles in maintaining the function of the cambium [16].

Stress response analysis showed that the transcript levels of *Ssu*-*sHsps* was generally induced by at least one tested stress, especially heat stress. The similar expression patterns of *sHsps* from different species in response to stress indicated that they might possess conservation function [9,10,13]. In order to analyze the potential regulatory mechanism, the *cis*-acting elements and the co-expression network of *Ssu-sHsps* were analyzed. As the binding sites of transcription factors, *cis*-acting elements in the promoter of the gene determine its expression patterns. In our study, a series of stress-, hormone-, and development-related *cis*-acting elements were detected in the promoter of *Ssu*-*sHsps.* HSE, the target site of Hsf, was abundantly present in the promoters of *Ssu*-*sHsps* (Figure 8). In our study, the *Hsf* family member (*HsfA6B* and *HsfA2*) were co-expressed with the C and CP subfamily *sHsps* (Figure 9). In *Arabidopsis*, *HsfA6B* participate in ABA-mediated salt and drought response and is required for heat stress resistance [36], and *HsfA2* is regarded as a heat-inducible transactivator that plays a positive role in enhancing the heat tolerance by activating the expression of *Hsp* or *sHsp* [37]. In addition, *DREB2C* was also detected in our co-expressed network and co-expressed with *sHsps* from the C and MT subfamily (Figure 9). In *Arabidopsis*, *DREB2C* is responsive to heat stress by activating the expression of *HsfA3* [38] and the *Thermotolerance-related Phytocystatin 4* (*AtCYS4*) [39], and *DREB2C* is also involved in ABA signing by regulating the expression of the ABA responsive gene [40]. In our study, several members in the C subfamily were induced by ABA treatment (Figure 6 and Figure 7), this infers that HsfA6B, HsfA2, and DREB2C may directly or indirectly bind to the promoter region of *Ssu*-*sHsps* to activate their expression in the ABA dependent pathway. In addition, some other transcription factors and functional genes that are related to stress response and development were identified in the co-expression network (Figure 9 and Table S2). This suggests that *Ssu*-*sHsps* might be involved in stress tolerance and plant development through cooperating with these genes. Further verification will need to be performed in the future.

## 4. Materials and Methods

### 4.1. Identification of sHsp Genes in S. suchowensis Genome

The protein sequence of *S. suchowensis* was obtained from the *S. suchowensis* genome database (http://115.29.234.170/cgi-bin/gbrowse/gbrowse/Ssuchowensis4/) [23]. The sHsp protein sequences of *A. thaliana* and *P. trichocarpa* obtained from phytozome (https://phytozome.jgi.doe.gov) were used as query sequences for the BLASTP to identify sHsp sequences in the *S. suchowensis* genome, with a cutoff *E*-value < 10^−10^. An additional search strategy, Hsp20 domain (PF00011) was used as a query to identify sHsp proteins using the HMMER (v3.0) software with an *E*-value < 10^−3^. SMART program (http://smart.embl-heidelberg.de/) [41] and the InterPro databases (http://www.ebi.ac.uk/interpro/) [42] were used to screen and confirm the Hsp20 domain in the candidate sHsp proteins, and the proteins lacking Hsp20 domain were removed.

### 4.2. Sequence Analysis and Structural Characterization

The isoelectric points (pI) and molecular weight (Mw) of Ssu-sHsps were estimated using the compute pI/Mw tool from ExPASy (http://web.expasy.org/compute_pi). The online program gene structure display server (http://www.gsds.cbi.pku.edu.cn/) [43] was used to analyze the gene structure (exon-intron) of *Ssu-sHsps*. The motifs in Ssu-sHsps were investigated using the MEME 4.11.1 online program (http://meme.nbcr.net/meme/intro.html) [44]. The protein subcellular localization was predicted using WoLF PSORT (http://psort.hgc.jp/) [45].

### 4.3. Phylogenetic Analysis and Chromosomal Location

The sHsp protein sequences from *S. suchowensis*, *A. thaliana,* and *P. trichocarpa* were used for phylogenetic analysis. Clustal X 2.1 software [46] was used for the multiple alignment of sHsp proteins and unrooted phylogenetic trees were constructed using MEGA 6.06 software [47] with the neighbor-joining method. The information of the chromosomal location of all *Ssu*-*sHsp* genes was downloaded from Phytozome 12.1 and the chromosomal locations and duplication of the *Ssu*-*sHsp* genes were drawn using Circos software [48]. In the analysis of the duplication, the array of two genes located within a 100 kb region were regarded as duplicated genes. DnaSP 5 software (http://www.ub.edu/dnasp/) [49] was used to calculate the ratio between nonsynonymous (*K*a) and synonymous nucleotide substitutions (*K*s).

### 4.4. In Silico Analysis of Regulatory Elements in the Promoter Regions of the Ssu-sHsp Gene Family

The upstream regions (2.0 kb) of the translation initiation sites (ATG) of *Ssu-sHsp* genes were used as promoter fragments. In addition, the elements were located in the promoter sequences using the program PlantCARE online (http://bioinformatics. psb.ugent.be/webtools/plantcare/html/) [50].

### 4.5. Plant Materials, Growth Conditions, and Treatments

The different tissues of cultivated *S. suchowensis,* including root (R), stem (S), leaf (L), female catkin (FC), and male catkin (MC), were harvested from triennial *S. suchowensis*. Four-week-old clones of *S. suchowensis* were water cultured using Hoagland’s nutrient solution in a growth chamber under long-day conditions (16 h light/8 h dark) at 25 °C/22 °C (day/night). In order to investigate the expression profiles of *Ssu*-*sHsps* under abiotic stresses and hormone treatment, the clones were treated under 150 mM NaCl (for salt stress), 20% (*w*/*v*) polyethylene glycol (PEG 6000, for drought stress), 37 °C (for heat stress), 4 °C (for cold stress), or 100 µM abscisic acid (ABA), respectively. Samples were collected after five time periods (0, 1, 6, 12, and 24 h) of each treatment. Fully matured leaves from five individual clones were immediately harvested at each time period of different treatments, and then rapidly frozen in liquid nitrogen and stored at −80 °C for further analysis. The dosages of the abiotic stresses and hormone treatment were determined based on treatments in *S. suchowensis* [22]. Three biological replicates were performed in different pots for each treatment.

### 4.6. RNA Isolation and qRT-PCR

The RNA isolation, primer design, qRT-PCR reactions, and program were performed according to Li et al [51]. Because of the high similarity of *Ssu*-*sHsp13*.*3*-*CI*, *Ssu*-*sHsp17*.*7A*-*CI*, *Ssu*-*sHsp17*.*7B*-*CI*, *Ssu*-*sHsp17*.*7C*-*CI,* and *Ssu*-*sHsp17*.*8*-*CI*, their expression levels cannot be distinguished by qRT-PCR. On the basis of our previous research, *EF1a* (*Elongation factor*-*1 alpha*) was used as reference gene for different tissue types; *HIS* (*Histone superfamily protein H3*) and *Actin7* for cold treatment and salt treatment, respectively; *UBC* (*Ubiquitin-conjugating enzyme E2*) both for heat and drought treatment [51]. The expression levels of *Ssu*-*sHsps* under abiotic stresses and hormone treatment were compared with control (0 h). In addition, the significantly induced genes were selected when the value of Log2 (fold change) ≥1. All the primers used in this study are listed in Table S3.

### 4.7. Integrated Network Analysis

The co-expression data of *sHsps* was obtained from Phytozome (https://phytozome.jgi.doe.gov/pz/portal.html). The co-expression network was constructed according to Tian et al [52].

## 5. Conclusions

On the basis of the *S. suchowensis* genome sequence, a total of 35 *Ssu-sHsp* genes were identified in this study. A comprehensive genome-wide analysis was performed to reveal phylogenetic relationship, gene organization, protein structure, chromosomal localization, co-expression network, and expression patterns across the different tissues and under stress conditions. According to the phylogenetic relationships and the predicted subcellular localization, the 35 *Ssu-sHsps* were classified into four subfamilies (C, CP, MT, and PX). A total of eight paralogous pairs were identified in the *Ssu-sHsp* gene family, five pairs were generated by tandem duplication and three pairs were generated by whole genome duplication, and all of them had undergone purifying selection during evolution. Multiple sequence alignment and motif analysis showed that the sequences of the Hsp20 domain were diverse among Ssu-sHsps. Expression profiling and co-expression network analyses revealed that *Ssu*-s*Hsps* might be involved in plant stress tolerance and development through cooperating with a series of transcription factors and functional genes. The results provide a basis for comprehensively understanding the *Ssu-sHsp* gene family and will be beneficial to investigate the function of *Ssu*-*sHsps*.

## Figures and Tables

**Table 1:** The *Ssu*-*sHsp* gene family identified in *S. suchowensis.*

Gene Name	Gene ID	Location (bp)	Coding Sequence (CDS) Length (bp)	Protein Length (aa)	Molecular Weight (MW) (kDa)/pI	Subfamily
*Ssu-sHsp13.3-CI*	willow_GLEAN_10013268	chr19:6883846-6884193(−)	348	116	13.3/8.92	CI
*Ssu-sHsp16.5-CI*	willow_GLEAN_10019104	chr01:18114468-18114899(−)	432	144	16.5/5.81	CI
*Ssu-sHsp17.7A-CI*	willow_GLEAN_10001930	scaffold704:2227-2685(−)	459	153	17.7/5.52	CI
*Ssu-sHsp17.7B-CI*	willow_GLEAN_10001931	scaffold704:5790-6248(−)	459	153	17.7/5.5	CI
*Ssu-sHsp17.7C-CI*	willow_GLEAN_10013270	chr19:6871309-6871767(−)	459	153	17.7/5.34	CI
*Ssu-sHsp17.8-CI*	willow_GLEAN_10013271	chr19:6867577-6868035(−)	459	153	17.8/5.72	CI
*Ssu-sHsp17.9-CI*	willow_GLEAN_10023007	chr09:3274010-3274480(−)	471	156	17.9/6.77	CI
*Ssu-sHsp18.0A-CI*	willow_GLEAN_10015047	chr01:12867685-12868164(−)	480	159	18.0/5.82	CI
*Ssu-sHsp18.0B-CI*	willow_GLEAN_10015393	chr04:10868237-10868704(−)	468	156	18.0/5.54	CI
*Ssu-sHsp18.1-CI*	willow_GLEAN_10023005	chr09:3270244-3270711(−)	468	156	18.1/6.77	CI
*Ssu-sHsp18.2A-CI*	willow_GLEAN_10001113	scaffold03801:634-1107(+)	474	158	18.2/5.55	CI
*Ssu-sHsp18.2B-CI*	willow_GLEAN_10022937	chr09:2679684-2680154(−)	471	157	18.2/6.76	CI
*Ssu-sHsp18.3-CI*	willow_GLEAN_10001192	scaffold03321:1968-2444(−)	477	159	18.3/5.51	CI
*Ssu-sHsp18.5-CI*	willow_GLEAN_10019417	chr10:11252074-11252562(+)	489	163	18.5/5.57	CI
*Ssu-sHsp18.6-CI*	willow_GLEAN_10024904	chr08:2910419-2910907(−)	489	163	18.6/6.77	CI
*Ssu-sHsp23.5-CI*	willow_GLEAN_10013269	chr19:6875448-6880158(−)	618	206	23.5/5.77	CI
*Ssu-sHsp17.8A-CII*	willow_GLEAN_10007257	chr15:3576323-3576796(−)	474	158	17.8/6.74	CII
*Ssu-sHsp17.8B-CII*	willow_GLEAN_10026164	chr06:13631015-13631488(+)	474	158	17.8/6.32	CII
*Ssu-sHsp17.8-CIII*	willow_GLEAN_10001482	scaffold02057:529-1140(−)	471	157	17.8/6.32	CIII
*Ssu-sHsp21.9-CIV*	willow_GLEAN_10001528	scaffold01874:2153-2906(−)	576	191	21.9/9.59	CIV
*Ssu-sHsp23.1-CIV*	willow_GLEAN_10008116	chr13:3276222-3276989(−)	615	204	23.1/9.65	CIV
*Ssu-sHsp28.0-CIV*	willow_GLEAN_10023903	chr09:8210641-8211561(−)	762	253	28.0/7.8	CIV
*Ssu-sHsp38.2-CIV*	willow_GLEAN_10015419	chr04:11093447-11096554(−)	1050	350	38.2/6.32	CIV
*Ssu-sHsp38.7-CIV*	willow_GLEAN_10015420	chr04:11103548-11105801(−)	1053	350	38.7/8.43	CIV
*Ssu-sHsp22.2-CV*	willow_GLEAN_10017670	chr01:24155089-24155990(+)	591	196	22.2/5.11	CV
*Ssu-sHsp23.0-CV*	willow_GLEAN_10006909	chr11:9175250-9176081(+)	609	202	23.0/5.02	CV
*Ssu-sHsp26.3-CP*	willow_GLEAN_10005204	chr12:637010-638195(+)	720	240	26.3/6.35	CP
*Ssu-sHsp26.5-CP*	willow_GLEAN_10014198	chr10:277029-277816(−)	705	235	26.5/7.89	CP
*Ssu-sHsp23.8-MT*	willow_GLEAN_10017874	chr16:5313895-5314699(−)	645	215	23.8/5.44	MT
*Ssu-sHsp25.6-MT*	willow_GLEAN_10022135	chr03:3924709-3926100(−)	678	225	25.6/5.82	MT
*Ssu-sHsp15.9A-PX*	willow_GLEAN_10001081	scaffold04006:1618-2317(+)	420	140	15.9/7.02	PX
*Ssu-sHsp15.9B-PX*	willow_GLEAN_10018481	chr04:3932697-3933402(+)	426	141	15.9/6.2	PX
*Ssu-sHsp16.1-PX*	willow_GLEAN_10002647	chr17:7449402-7450136(+)	426	142	16.1/6.92	PX
*Ssu-sHsp23.1*	willow_GLEAN_10011114	chr15:5028375-5029436(+)	609	202	23.1/4.6	*
*Ssu-sHsp31.8*	willow_GLEAN_10023904	chr09:8208040-8209110(−)	858	285	31.8/6.04	*

C, cytosol; CP, chloroplast; PX, peroxisome; MT, mitochondria; *, unclustered member.

**Table 2:** Divergence between paralogous *Ssu*-*sHsp* gene pairs in *S. suchowensis.*

Gene 1	Gene 2	*K*a	*K*s	*K*a/*K*s	Duplication	Subfamily	*p*-Value (Fisher)
*Ssu*-*sHsp17*.*7A*-*CI*	*Ssu*-*sHsp17*.7*B*-*CI*	0.0167	0.0676	0.2475	T	CI	4.9063 × 10^−3^
S*su*-*sHsp17*.*8*-*CI*	*Ssu*-*sHsp17*.*7C*-*CI*	0.0349	0.0612	0.5697	T	CI	1.4443 × 10^−1^
*Ssu*-*sHsp18*.*1*-*CI*	*Ssu*-*sHsp17*.*9*-*CI*	0.0061	0.0477	0.1271	T	CI	1.0528 × 10^−3^
*Ssu-sHsp23*.*5*-*CI*	*Ssu*-*sHsp13*.*3*-*CI*	0.0185	0.0485	0.3808	T	CI	5.1005 × 10^−2^
*Ssu*-*sHsp18*.*6*-*CI*	*Ssu-sHsp18*.*5*-*CI*	0.0711	0.4235	0.1678	W	CI	2.9675 × 10^−17^
*Ssu*-*sHsp38*.*2*-*CIV*	*Ssu*-*sHsp38*.*7*-*CIV*	0.1624	0.1997	0.8135	T	CIV	1.8343 × 10^−1^
*Ssu*-*sHsp22*.*2*-*CV*	*Ssu*-*sHsp23*.0-*CV*	0.1153	0.5552	0.2077	W	CV	1.6456 × 10^−24^
*Ssu*-*sHsp25*.*6*-*MT*	*Ssu*-*sHsp23*.*8*-*MT*	0.5873	0.9437	0.6223	W	MT	3.8210 × 10^−17^

## Supplementary Materials

Supplementary materials can be found at http://www.mdpi.com/1422-0067/19/10/3246/s1.
