# Supplementary figures and images for: Genome-Wide Characterization of the sHsp Gene Family in Salix suchowensis Reveals Its Functions under Different Abiotic Stresses
Source: Int J Mol Sci. 2018 Oct 19;19(10):3246. doi: 10.3390/ijms19103246 (PMC6214038; doi:10.3390/ijms19103246)

**
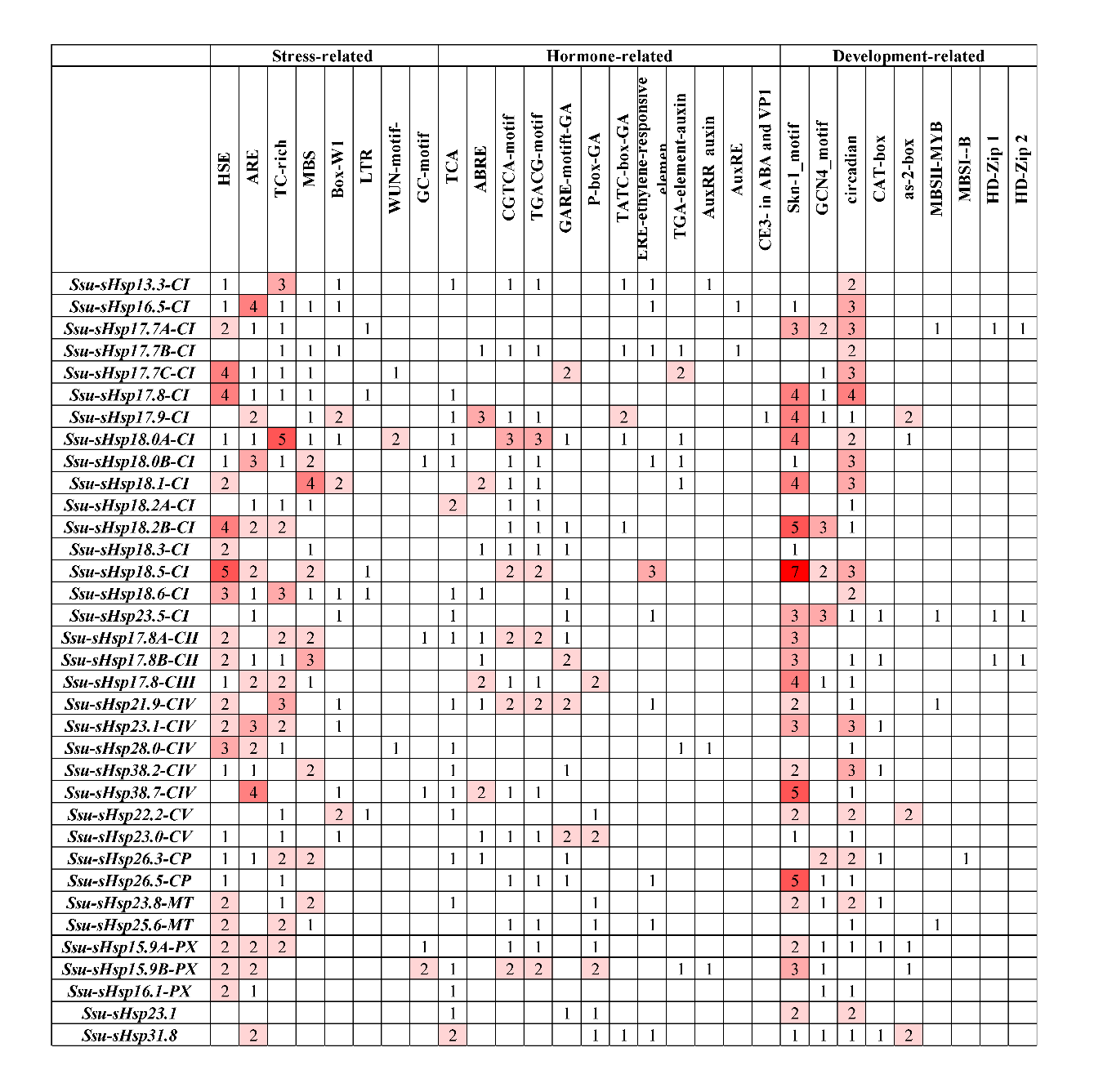
Figure S1.** The number of each *cis*-acting element in the promoter region of each *Ssu-sHsp* gene.

Supplement: Supplementary file 1 [file ijms-19-03246-s001.zip › ijms-368794-supplementary-final check/Supplementary Figure S1.docx]
